# Delayed skeletal muscle repair following inflammatory damage in simulated agent-based models of muscle regeneration

**DOI:** 10.1371/journal.pcbi.1011042

**Published:** 2023-04-06

**Authors:** Stephanie Khuu, Justin W. Fernandez, Geoffrey G. Handsfield

**Affiliations:** 1 Auckland Bioengineering Institute, The University of Auckland, Auckland, New Zealand; 2 Department of Engineering Science, The University of Auckland, Auckland, New Zealand; University of Virginia, UNITED STATES

## Abstract

Healthy skeletal muscle undergoes repair in response to mechanically localised strains during activities such as exercise. The ability of cells to transduce the external stimuli into a cascade of cell signalling responses is important to the process of muscle repair and regeneration. In chronic myopathies such as Duchenne muscular dystrophy and inflammatory myopathies, muscle is often subject to chronic necrosis and inflammation that perturbs tissue homeostasis and leads to non-localised, widespread damage across the tissue. Here we present an agent-based model that simulates muscle repair in response to both localised eccentric contractions similar to what would be experienced during exercise, and non-localised widespread inflammatory damage that is present in chronic disease. Computational modelling of muscle repair allows for *in silico* exploration of phenomena related to muscle disease. In our model, widespread inflammation led to delayed clearance of tissue damage, and delayed repair for recovery of initial fibril counts at all damage levels. Macrophage recruitment was delayed and significantly higher in widespread compared to localised damage. At higher damage percentages of 10%, widespread damage led to impaired muscle regeneration and changes in muscle geometry that represented alterations commonly observed in chronic myopathies, such as fibrosis. This computational work offers insight into the progression and aetiology of inflammatory muscle diseases, and suggests a focus on the muscle regeneration cascade in understanding the progression of muscle damage in inflammatory myopathies.

## Author summary

Skeletal muscles are robust tissues that allow individuals to move and perform daily activities. Healthy muscle can repair following exercise and injury within 2–4 weeks, depending on severity. The damage to skeletal muscle following exercise is typically localised to areas along muscle fibres; however, in muscles affected by Duchenne Muscular Dystrophy and inflammatory myopathies, the damage to muscle tissue is more widespread. Little is known about how the localisation of injury in muscle affects the response of cells and chemicals involved in the repair process. Here, we built a computational model of human skeletal muscle repair following both localised (typical) and widespread (inflammatory) injury to explore the effect of injury localisation on repair time and cell numbers. Simulations of widespread damage similar to that found in pathological scenarios led to delayed clearance of damaged tissue and delayed repair of the initial fibril number. These changes led to morphological changes in muscle geometry and reflected commonly observed changes in pathological muscle.

## Introduction

Muscle repair is often described as a linear series of specific events at the cellular level, but in fact the muscle cell environment, and therefore muscle repair environment, is multifaceted with dynamic interactions between cell populations, their chemical environment, and external stimuli. *In silico* mechanobiological modelling can be used to simulate the process of muscle damage and repair, from the mechanical loading of the tissue to the subsequent changes to the system during repair. These models can be used to test individual variables that affect muscle repair, in ways that may exceed what is experimentally feasible. Agent-based modelling (ABM) is a technique well-suited to the exploration of biological questions pertaining to the process of skeletal muscle regeneration [1,2]. In ABM, dynamic interactions reflect the complex and nonlinear nature of physiological systems, and often lead to emergent phenomena. Previously ABM has been used to explore disuse induced atrophy [3], the Duchenne muscular dystrophy (DMD) muscle microenvironment [4], and the muscle repair process in cerebral palsy [5].

Here we have created an agent-based mechanobiological model of human skeletal muscle that models cell populations from the skeletal muscle environment as agents on a 2D grid, to explore whether muscle fibre bundles subjected to localised strain (akin to exercise-induced damage) undergo repair differently than muscle subjected to non-localised widespread damage (akin to inflammatory damage). Agents in the model include muscle fibres composed of fibrils, satellite cells (SCs), macrophages, neutrophils, extracellular matrix (ECM), and fibroblasts. Extracellular guidance cues include cytokines and growth factors (TNF-α, IL-6, TGF-β, HGF, IL-10, IL-15, IGF-1) that guide cell behaviour. The ABM is used to simulate repair following a bout of localised versus widespread damage. Localised strain was simulated using mechanical data from a finite element model of a muscle fibre bundle with the same geometry as the ABM. This workflow was created to investigate the differences in simulation outcomes such as fibril recovery, fibre morphology, and damage clearance time, between localised and widespread damage. The finding that muscle repair proceeds differently in localised versus widespread damage has implications for chronic myopathies such as DMD and inflammatory myopathies (IMs) that are characterised by prolonged inflammatory cell invasion, progressive weakening, loss of muscle mass, and necrosis that overwhelms the muscle repair response [6–8]. The prolonged inflammation experienced by skeletal muscle in chronic myopathies leads to diffuse widespread damage compared to the damage caused by exercise-induced damage alone. The model presented here is used to compare cell populations and chemical concentrations throughout the cycle of repair in both localised and widespread randomised damage, the outcomes of which are used to infer the implications of widespread inflammatory damage on the process of muscle repair. This modelling approach can thus provide insight on how the type of injury influences muscle repair, and may elucidate the aetiology of inflammatory muscle diseases while emphasising the importance of mechanical signals in transducing healthy muscle repair.

Skeletal muscle undergoes remodelling in response to its use and the demands placed upon it [9]. Individual muscle fibres grow in size by adding myonuclei and associated cytoplasm to existing fibres [10]. The contraction of these muscle fibres via cross-bridge cycling allows movement of joints, and muscle fibre hypertrophy is an advantageous adaptation that, at the macro-scale, increases the maximum strength of the muscle around that joint [11]. Hypertrophy is elicited by increased and repeated bouts of muscle loading, particularly eccentric loading [12–14]. Muscle loading causes mechanical damage to the cells and tissue, which is transduced into a cascade of chemical changes that affects the repair and regeneration of damaged tissue [14,15]. This well-regulated process involves muscle fibres, SCs, fibroblasts, inflammatory cells such as neutrophils and macrophages, the ECM, cytokines, and growth factors (TNF-α, IL-6, TGF-β, HGF, IL-10, IL-15, IGF-1) that guide cell behaviour [16–19].

Eccentric exercises cause localised areas of high strain on muscle fibres [20,21]. These exercises are also associated with higher levels of regeneration activity, highlighting the importance of directed mechanical insult as a step in the normal regeneration process. As a consequence of mechanically localised strain, cell activity is directed to specific repair sites that allow for timely clearance of damage and subsequent rebuilding of functional contractile muscle fibrils [22–24]. In juxtaposition to this is non-localised damage where cells do not transduce an external mechanical signal, and instead respond to widespread damage across the tissue, such as in chronic muscle disease [25,26]. The implications of this form of widespread inflammatory damage on the process of muscle regeneration have not been explored.

In the case of localised damage, high strains imposed on muscle fibre membranes disrupt and break the membrane, triggering a calcium imbalance [27]. In addition, mechanical stretching of skeletal muscle fibres increases extracellular HGF, and initiates an acute inflammatory response [28,29]. In the first 24 h following loading it is conventionally understood that neutrophils invade and begin to clear damaged tissues [17]. At 48–72 h, proinflammatory macrophages reach peak numbers in order to phagocytose debris before anti-inflammatory macrophages dominate, and their growth factors encourage the proliferation of regenerative SCs [24,30]. SCs are commonly studied muscle stem cells that are responsible for the repair of damaged muscle tissue [9]. SCs become active in the presence of HGF [31] and proliferate in the presence of cytokines such as IL-6 and IGF-1 [32–35], before differentiating to myoblasts and fusing to existing fibres [36]. Fibroblasts, on the other hand, are responsible for remodelling the ECM—a component of muscle tissue essential for efficient force transfer across the entire muscle [37]. The repair process typically takes 2–4 weeks [27]; however, it is unknown how the process may be impacted by non-localised or widespread damage that is not due to mechanical transduction. The present study evaluates whether simulations of localised clustered pixel damage of skeletal muscle have an altered repair time course compared to non-localised widespread damage of the same magnitude, that is represented as randomly dispersed damaged pixels across the tissue. Agent-based models were used to simulate the differences in damage clearance, repair timeframe, and changes to muscle fibre morphology between regeneration from widespread inflammatory versus localised damage.

## Methods

**Fig 1:**
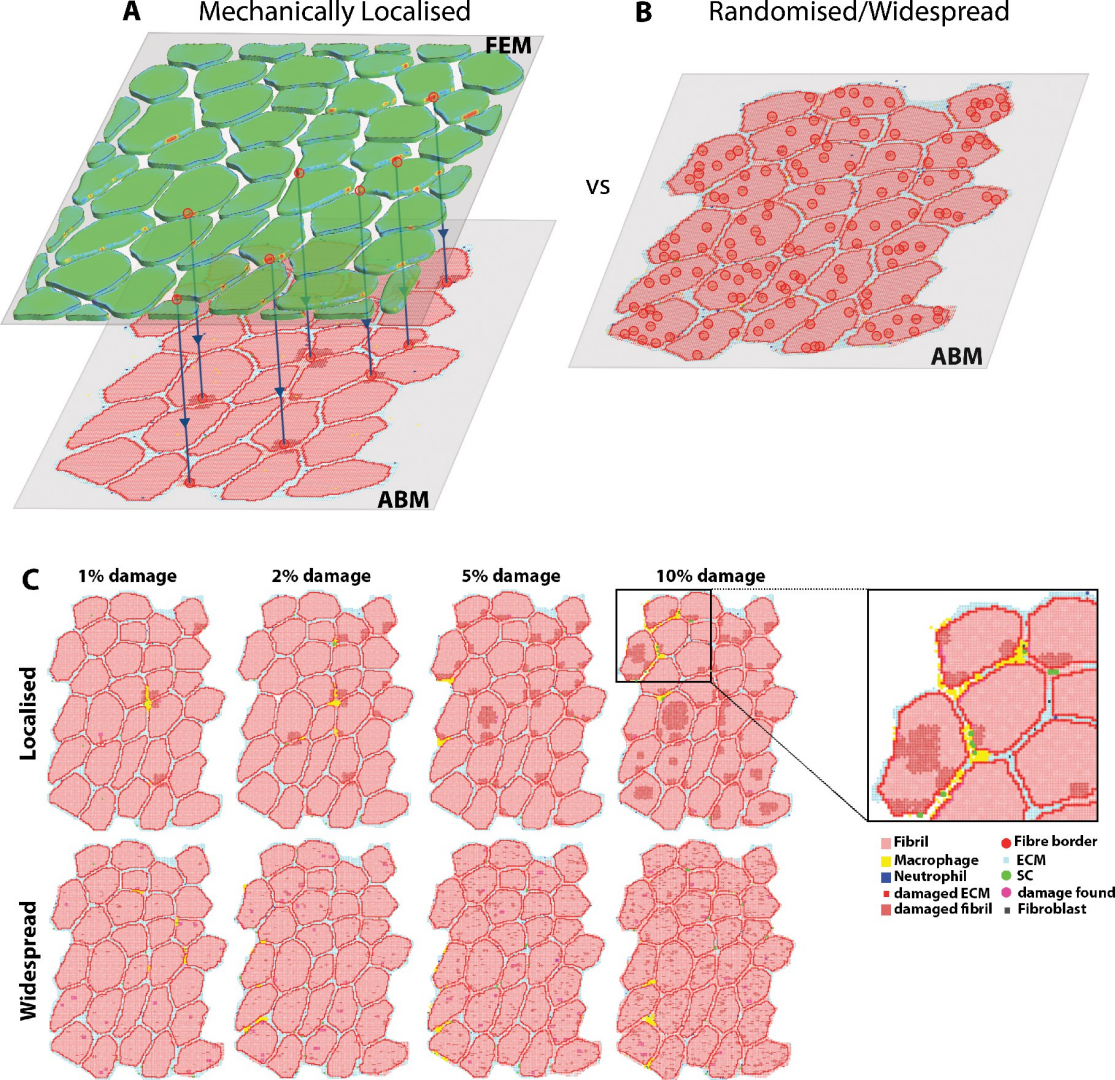
Methods of strain input for the agent-based model. (A) Mechanically localised strain values from FEM of a muscle bundle were used to seed an ABM of muscle damage. This was compared to (B) widespread damage seeded by selecting random muscle fibrils in the ABM and labelling them as damaged in the simulation. (C) Visualisation of the damage in localised vs widespread agent-based simulations, zoom inset displays the agents used in this ABM.

We created an agent-based model of typical muscle regeneration with mechanical strain data from a finite element model (FEM) using data from experimental studies in the literature [9,24,31,38–40]. For localised damage, the workflow began with an FEM of a muscle fibre bundle undergoing active eccentric contraction, where the highest 10% of all strain values were taken from the FEM output and used to seed locations of damage in the ABM (Fig 1A). For the widespread damage model, no FEM data was used, and locations of damage were randomly assigned across the grid (Fig 1B).

### ABM and FEM geometry

Initial geometry of the models was taken from images of a single human vastus lateralis muscle cross-section consisting of 30 fibres [39]. We segmented the fibres and ECM using k-means clustering in the MATLAB Statistics and Machine Learning Toolbox (The MathWorks, Inc., Natick, MA). The resulting 2D ECM and fibre pixels were used to 1) create the geometry for an ABM by directly seeding fibril pixels at the corresponding coordinate points, and 2) create FEM geometry by extruding 2D fibre and ECM borders into 3D geometries. FEM development has been described in detail previously [5], with the only two differences being the fibre geometry (now with 30 fibres) and the displacement of fibres set to 10% rather than 20%. Briefly, the same muscle cross section used for the ABM was used to create an FE geometry of 30 muscle fibres in a bundle in FEBio [41], and simulated active eccentric contraction with 10% displacement, to determine locations of high strain and mechanical damage. The coordinate points that had the highest 10% of strain values were then imported to the ABM to guide cell behaviour. Since the same geometry was used to build both models, the strain distribution from the FEM could be registered to the matching coordinates in the ABM geometry. FEM results can be found in the github repository at https://github.com/stkhuu/muscleRegen/blob/main/20210730_03newGeo00015_BC.log.

### Implementation of damage

The ABMs simulated either widespread or localised damage and represented muscle regeneration over 28 days following prescribed damage levels of 0, 1, 2, 5 and 10%. Localised damage was implemented by seeding the highest 0, 1, 2, 5 or 10% of strain values from the FEM (Fig 1A) onto the equivalent ABM coordinate points, which then marked corresponding fibrils as ‘damaged’, in a binary fashion. Widespread damage was implemented by randomly labelling 0, 1, 2, 5 or 10% of initial fibrils as damaged throughout the cross-section of the muscle (Fig 1B). Agents are not programmed to respond directly to mechanical inputs; however, the mechanical stimulus does impact the location of cytokine release in the ABM.

### Agent-based modelling

**Fig 2:**
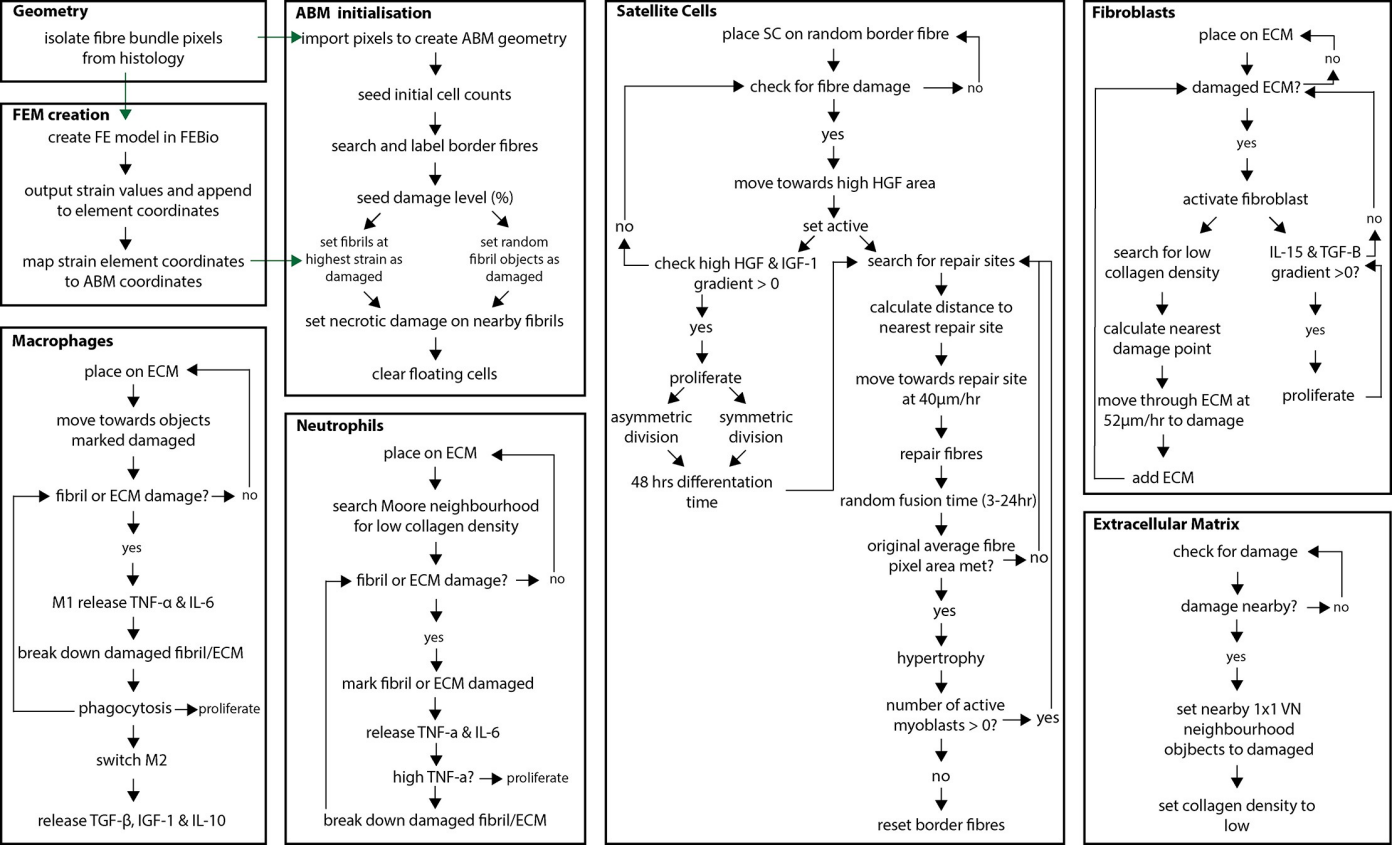
Flowchart of ABM agent behaviour. Green arrows indicate where data was imported to seed the ABM. Strain values from the FEM were taken in the case of localised damage, while random fibril objects were set to ‘damaged’ for the widespread damage simulations.

The ABM was developed in Repast Simphony [42], a Java-based modelling toolkit, and expands on ABM previously described in Khuu et al. [5]. ABM geometry was based on pixel geometry of a histological slice from the literature [39] (see aforementioned description). Pixels were then imported onto a grid at the corresponding coordinate points in Repast Simphony. Each pixel represents 3.61 μm^2^ of the muscle fibre environment. Each of the 30 muscle fibres in the ABM represented a cross-sectional slice thickness of 50 μm. The ABM rules were developed based on literature descriptions of interactions between muscle fibres, non-fibre cells, and their chemical environment; agent actions and interactions are described in detail in the following subsections, as well as in Fig 2. Agents comprised muscle fibres, macrophages, neutrophils, SCs, fibroblasts, and ECM. Cytokines and growth factors were treated as non-cellular environmental factors in the model and thus influenced the behaviour of agents. These extracellular guidance cues included IL-10, IL-15, IL-6, IGF-1, TGF-β, HGF, and TNF-α. Scaling coefficients for the chemical factors were optimised using the genetic algorithm method described below. Initial ABM geometry consisted of 25840 fibril elements and 4318 ECM elements on the 2-D grid. Each time step represented one hour of muscle regeneration; cell speeds were adjusted according to time step and pixel area. Each simulation was run 50 times, and simulation results are shown as mean ± standard deviation (SD). The ABM framework is based on [5] with changes to fibre geometry, input strain thresholds to match the current FEM strain values, fibroblast cell migration, SC movement to repair the next nearest damaged neighbour, and cytokine and growth factor expression coefficients determined using an optimisation algorithm (see subsection Genetic Algorithm). The complete model code, portable archive file, and instructions (readme file) for running the model can be found at https://github.com/stkhuu/muscleRegen.git.

### Fibrils & ECM

Fibrils and ECM agents were seeded based on k-means clustering of pixels from our source histological image [39]. Geometry was recreated by placing fibre and ECM pixels at their corresponding coordinates on the 2D ABM grid. Fibrils were damaged using either the localised or random method. Localised damage used the coordinates of the highest strain values to set fibrils to ‘damaged’. The random damage method involved setting agents at random coordinate points within a fibre to a damaged state. Damage levels were set according to the user prescribed levels for each model. Once damaged, surrounding fibrils and ECM also underwent necrosis if within the immediate (3x3) Von Neumann neighbourhood of the damaged fibril. The collagen density of the ECM decreased as inflammatory cells cleared the damaged tissue and was reset when the ECM was repaired.

### Neutrophils & macrophages

Thirty macrophages and 30 neutrophil agents were initialised to random grid cells that contained ECM agents. The seeding densities of agents was set to 30 to 1) stabilise the simulation and 2) reach peak amplitude at times that are in accordance with the literature. Lower seeding densities lead to unstable simulations that did not run to completion. In the absence of prescribed damage, these agents do not move or proliferate. Both agent types could move from one grid point to another per timestep under both simulation conditions. Neutrophils searched a Moore neighbourhood for points of low collagen density to which to move. If the agent came across damaged fibrils or ECM, it would release pro-inflammatory cytokines TNF-α and IL-6 [43–45]. Neutrophil and macrophage agents that encountered damaged ECM or fibrils then underwent proliferation to areas of high TNF- α and IL-6 concentration. Neutrophils typically peak within 24hrs [46]. When a fibre or ECM agent was sufficiently broken down, neutrophils or macrophages phagocytosed the debris [17]. M1 macrophages agents initially released pro-inflammatory TNF-α and IL-6 and were programmed to proliferate when there was persistent fibre damage [47]. Once macrophage agents had phagocytosed one or more damaged fibre or ECM components in the model, they switched to an anti-inflammatory or M2 phenotype [30,48], secreting anti-inflammatory cytokines that allow satellite cell proliferation [30]. For modelling purposes, the M1 and M2 subtypes were represented by one agent population that was able to transition from pro-inflammatory state to the other. M1 and M2 macrophages were present in the simulation at the same time. These agents were not prescribed a speed and instead moved to damage locations once per time step, and it was assumed that they could move across more than a single pixel length within the hour. Macrophage and neutrophil numbers have been compared to literature values in Khuu et al [5], where days post injury for peak amplitude of cell counts was consistent with existing studies. Objects that were phagocytosed included ECM and fibrils. Fibrils needed to be identified by macrophages and then took 2 h to phagocytose. ECM objects required 4 h. This was based on tuning the time for peak concentration of macrophages and neutrophils to the literature [49]. When agents were phagocytosed, they were removed in binary fashion, i.e. the object was removed from the simulation grid.

### Satellite cells

SCs were seeded based on the assumption of 0.08 SC per fibre for 10 μm thick sections, and multiplied by five to suit a 50 μm thick cross-section [9,19]. For 30 fibres, the initial count was 12. SCs were placed at border fibre coordinates to replicate the muscle fibre niche. SC agents were programmed to activate in the presence of HGF and move towards the damaged areas, through both fibre and ECM media, based on the simulated cytokine gradient [50]. SC agents divided when levels of HGF remained high and IGF-1 was present [31,51]. Proliferation of SCs mimicked both asymmetric and symmetric division [52], with a 50% chance of either occurring in the model. SCs became active after three days and searched for sites needing repair. There was no prescribed maximum for SC number. SC speed was roughly 40 μm/h [53]. SCs could move across one pixel every 1/11^th^ of a time step based on speed and pixel area representation. Previous models [5] have matched SC peak amplitude timing to literature values and the same dynamics were used in the current model. Active SCs differentiated into myoblasts when there was a positive gradient of IGF-1 present compared to previous time steps. Myoblast fusion time represents the alignment, adherence and fusion of the myoblast to the damaged fibre. This process takes between 12–48 h [36,54]. Once SCs arrived at a repair site, a random number generator was run to assign a whole number between 3–24 from a uniform distribution, which assigned fusion time for the repair. SCs remained at the site until the number of time steps was greater than the random number. Once incorporated into the fibre, the SC agent is removed from the simulation and a fibril agent is added in its place. If the initial average fibre size was exceeded, the borders of fibres expanded, and hypertrophy occurred. When active SC number was zero, the simulation checked for fibrils that neighboured ECM components and labelled these as fibre borders to create a remodelled fibre outline where fibroblasts could then deposit collagen.

### Fibroblasts

Fibroblasts were seeded according to Mackey et al. [19] with initial count of 21 and maximum count of 67. Time course data for fibroblast cell counts were used to optimise cytokine levels (see below) by matching the simulated fibroblast counts with experimentally observed counts at 0, 2, 7 and 30 days post injury. Fibroblasts were assigned locations on the ECM randomly. Fibroblasts divided in the presence of a positive IL-15 and TGF-β gradient [55,56]. Fibroblast speed was set to 52 μm/h [57] and fibroblasts were restricted to move only through ECM components. Fibroblasts were programmed to find and travel towards ECM sites that needed remodelling. Fibroblasts laid down collagen to repair the ECM at these sites before searching for the next nearest empty ECM site. Collagen density of each point was reset after the ECM was repaired.

### Secreted factors

ABM cytokine and growth factor interactions are shown (Table 1) based on literature descriptions of growth factor and cytokine interactions. Growth factors and cytokines were represented by seven equations based on these interactions (Eqs 1–7). Global optimisation was used to determine 31 coefficients for the equations.

**Table 1:** Interactions between selected cytokines and growth factors involved in the muscle repair process. The effect of cytokines in the first column on the cytokines on the subsequent columns, e.g., IGF-1 decreases TNF-a levels in the first row. + denotes up-regulation and–denotes down-regulation as based on the listed references.

Cytokines	Regulatory response to cytokines						
	IGF-1	TNF-α	IL-6	HGF	IL-10	IL-15	TGF-β
**IGF-1**		**–[58]**					
**TNF-α**	**–[59,60]**		**+[40]**			**+[61]**	
**IL-6**	**–[58]**	**–[62]**		**+[62]**	**+[45]**		**+[63]**
**HGF**			**–[64]**		**+[28,64]**		
**IL-10**	**+[65]**	**–[65,66]**					
**IL-15**							**–[55]**
**TGF-β**	**–[67]**	**–[68]**	**–[69]**			**–[70]**	

### Genetic algorithm

**Fig 3:**
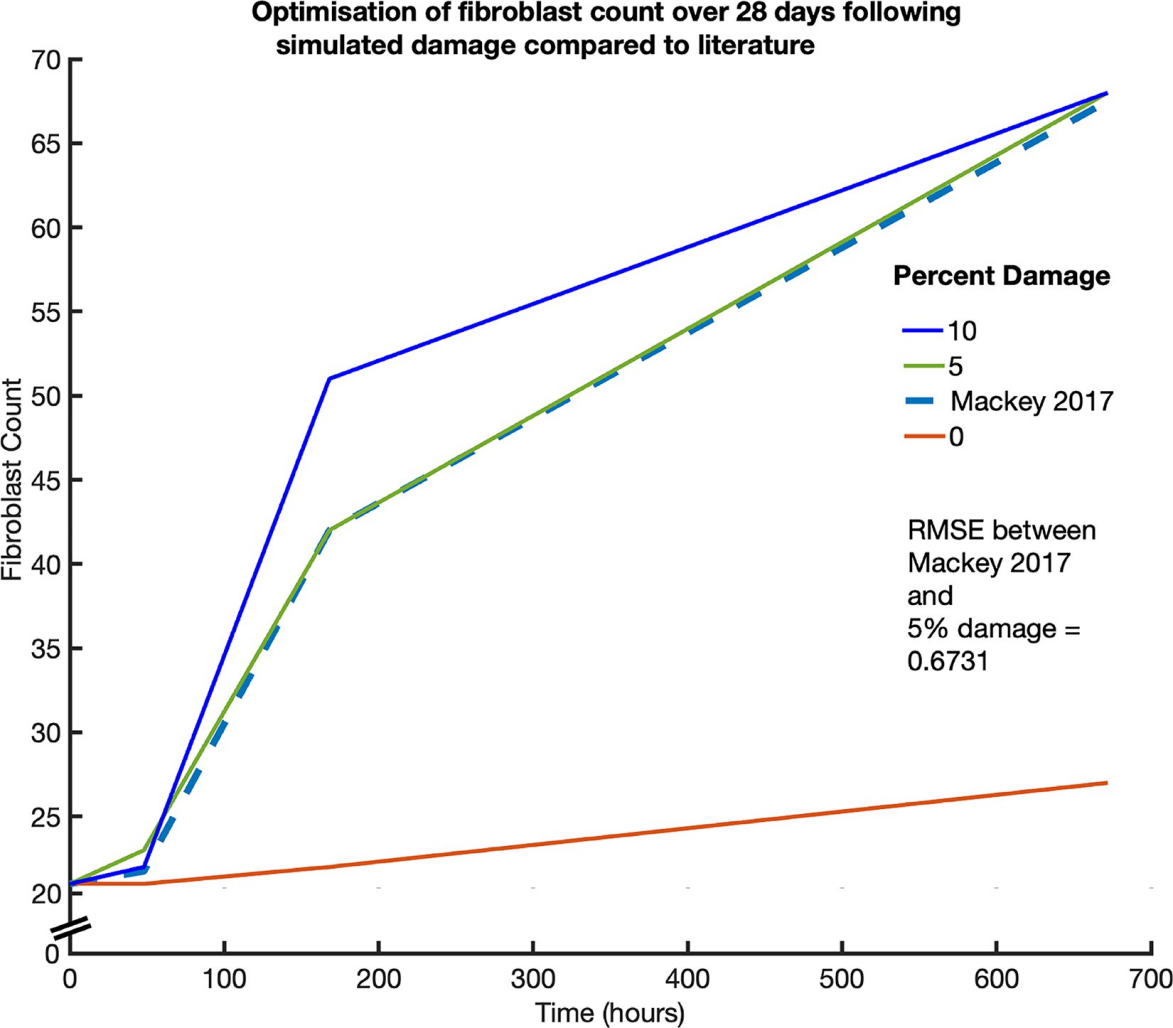
Simulation fibroblast count compared to reference fibroblast count at 0, 48, 168 and 672 hours post exercise induced injury.

The genetic algorithm (GA) optimisation method was used from the MATLAB global optimisation toolbox (The MathWorks, Inc), to minimise the objective function, in this case the RMSE between agent-based simulation outputs of fibroblast counts over time, and the known literature data of fibroblast counts over time [19]. Fibroblast numbers were used due to the availability of a dataset for healthy trained human muscle over 30 days, and their interactions with satellite cells. The reference dataset was for fibroblasts at 0, 48, 168 and 672 h post-exercise induced muscle repair. We optimised 31 cytokine coefficients corresponding to trends found in the literature (see Table 1) to produce fibroblast values similar to that of the reference dataset. The lower bound was set to 0 for all coefficients and the upper bound set to one for the first 25 coefficients, and 0.5 for the remaining six due to the high amplitude of the outputs when these values were set to greater values. The optimisation was run at least 150 times at 5% damage with new values seeded if the RMSE was lower than the previous run. Simulated fibroblast results were plotted against the reference dataset to calculate RMSE. Final RMSE was 0.6731 (Fig 3).

Specific optimisation code can be accessed at https://github.com/stkhuu/muscleRegen.git.


$$\frac{dIL10}{dt}=0.899\times AM+0.827\times IL6+0.670\times HGF$$ (1)

$$\frac{dIL15}{dt}=0.004\times F+0.558\times Mb+0.377\times TNF\alpha -0.825\times TGF\beta$$ (2)

$$\frac{dTNF}{dt}=0.273\times IM+0.456\times N+0.493\times Fb-0.346\times TGF\beta -0.106\times IL6-0.044\times IL10-0.485\times IGF1$$ (3)

$$\frac{dHGF}{dt}=0.755\times F+0.156\times IL6$$ (4)

$$\frac{dIL6}{dt}=0.043\times N+0.395\times IM+0.077\times F-0.077\times HGF-0.132\times TGF\beta +0.482\times TNF\alpha$$ (5)

$$\frac{dIGF1}{dt}=0.258\times AM+0.009\times F-0.788\times TNF\alpha -0.019\times TGF\beta -0.477\times IL6+0.826\times IL10$$ (6)

$$\frac{dTGFb}{dt}=0.007\times F+0.211\times AM+0.798\times IL6$$ (7)

Where *AM* was the number of anti-inflammatory macrophages, *N* was the number of neutrophils, *IM* was the number of pro-inflammatory macrophages, *Fb* was the number of fibroblasts, *F* was the number of fibres, and *Mb* was the number of myoblasts. All coefficients for Eqs 1–7 were seeded using the optimisation technique previously mentioned and determine both the starting condition and the concentration over time. In this way, the GA was used to determine the amount of cytokine production by cell types over time in the ABM.

To model secreted factors, we used the built-in Repast Simphony function ‘ValueLayerDiffuser’. The secreted factors exist on a grid superimposed on the agent grid, with their values accessible at every coordinate point by the agents. The ValueLayerDiffuser has an evaporation constant and a diffusion constant such that the edge of the space is considered a “value sink”, meaning any values making contact with the edge are evacuated out of the space. ‘Diffuse’ function then runs the diffusion with the current rates and values. It is described by Eq 8:

newValue=evap(ownValue+diffConst*(nghAvg−ownValue)) (8)

where *evap* is the evaporation constant, *ownValue* is the current value for the current cell, *diffConst* is the set diffusion constant value, and *nghAvg* is the weighted average of a cell’s neighbours. In this model the evaporation constant was set to 0.9, and the diffusion constant set to 0.1. These constants were set heuristically to generate cytokine removal that did not persist too long at a single grid point.

## Results

**Fig 4:**
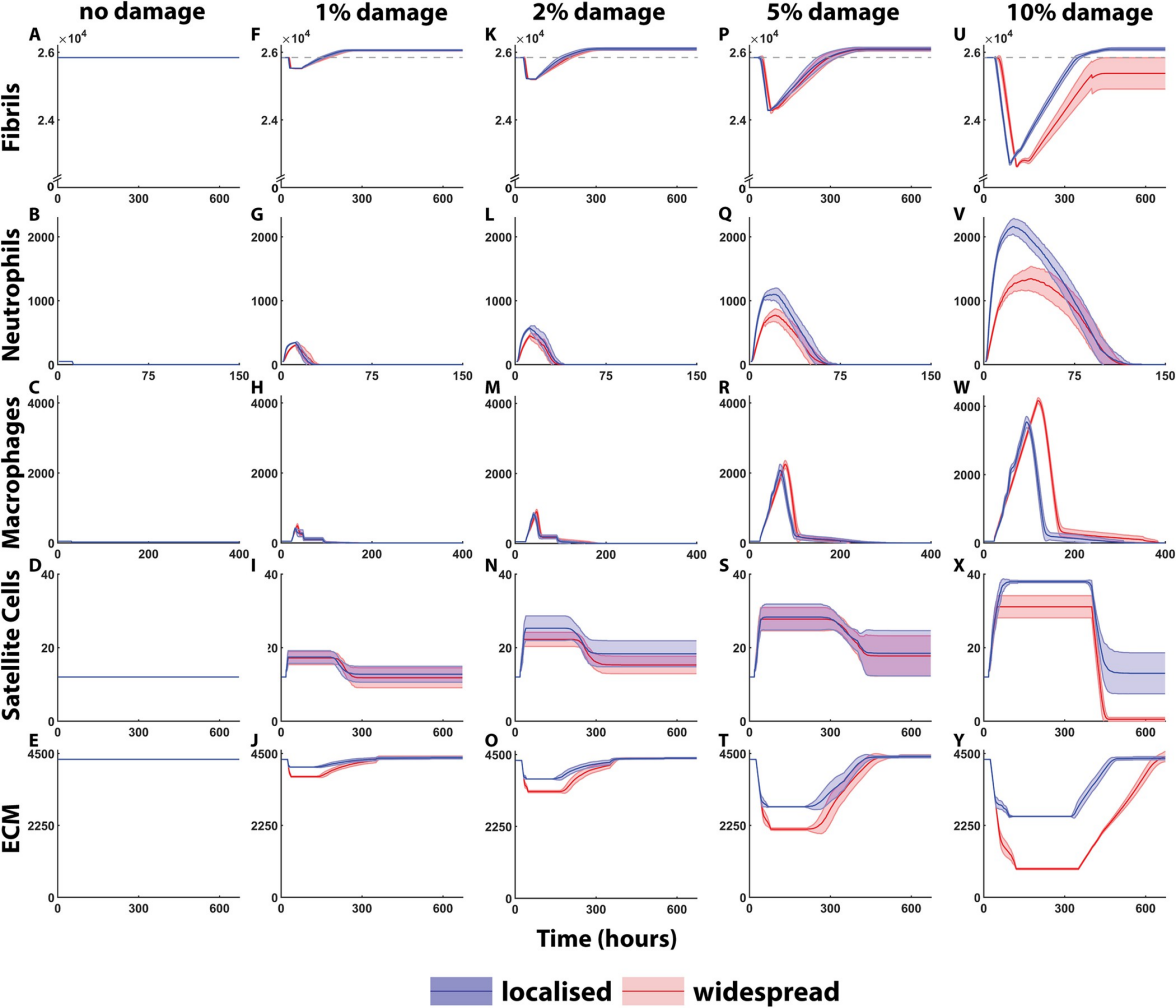
Cell counts (mean ± SD) over 672 h, from 50 simulations in localised and widespread damage conditions. (A-E) are control cell counts where no damage was imposed. (F-J) show fibril, neutrophil, macrophage, SC and ECM counts simulated over 672 h following 1% damage. (K-O) show 2% damage, (P-T) show 5% damage, and (U-Y) show 10% damage. Fibril repair is delayed and does not meet pre-injury levels at 10%. Neutrophils are increased in localised simulations compared to widespread. Macrophage recruitment is delayed and increased in widespread damage simulations. Satellite cell recruitment increases with damage level. The ECM count is recovered under both simulation conditions.

Cell counts over time were recorded from 50 simulations to understand the effect of localised and widespread damage on the muscle repair process. ‘No damage’ simulations showed no change in cell counts (Fig 4A–4E), as expected. Fibril counts for both damage scenarios were similar at the 1% damage level (Fig 4) throughout the simulated 672 h. The mean time to recovery (25840 fibrils) was t = 152 h for localised and t = 165 h for widespread at 1% damage, t = 176 h for localised and t = 199 h for widespread at 2% damage, and 290 h for localised and 296 h for widespread at 5% damage. In addition, damaged fibril removal was delayed in the 5% damage simulation. End point fibril counts for 2% and 5% damage were similar between localised [26100 ± 30 and 26100 ± 50 (mean ± SD) respectively], compared to widespread damage levels [26080 ± 40 and 26080 ± 20 (mean ± SD)], for 2% and 5% conditions, respectively. Damage of 10% introduced differences in fibril recovery with delayed clearance of damaged fibrils (t = 96 h for localised, t = 122 h for widespread). 10% localised damage fibril levels were repaired by t = 351 h while widespread damage was, on average, unable to reach initial fibril count in the simulation time frame. Endpoint fibril count was greater than initial in 10% localised [26090 ± 50 (mean ± SD)] but decreased following widespread damage [25370 ± 460 (mean ± SD)].

Peak neutrophil numbers were significantly greater in all localised simulations compared to widespread (Fig 4B, 4G, 4L, 4Q and 4V). Amplitude of neutrophil count scaled with prescribed damage percentage. Neutrophils peaked at 12 h, following 1 and 2% damage, and at 22 h in response to 5% damage. Following 10% damage, neutrophils peaked earlier in the localised (24 h) compared to widespread (39 h) simulations. Difference in neutrophil recruitment was most apparent at 10% prescribed damage with 2161 ± 121 (localised) and 1345 ± 193 (widespread) peak concentration. Contrastingly, macrophage counts were greater for widespread damage levels as opposed to localised (Fig 4C, 4H, 4M, 4R and 4W). Peak macrophage counts were delayed in widespread simulations, with the most obvious difference in the 10% simulations, with a delay of 27 h for widespread compared to localised and counts of 4157 ± 91 (widespread) and 3536 ± 34 (localised).

Mean satellite cell recruitment scaled with damage percentage. Following 1% damage, both conditions activated, on average, 18 SCs. At 2%, the mean SC activation was higher for localised (25) compared to widespread (21). Damage of 5% showed no significant differences in activation for localised damage compared to widespread (28 vs 27), whereas, during 10% damage repair, mean numbers for localised damage were much higher than widespread (37 SC in localised and 31 SC in widespread). 10% widespread damage also led to depletion of the SC pool at the end of the simulation.

**Fig 5:**
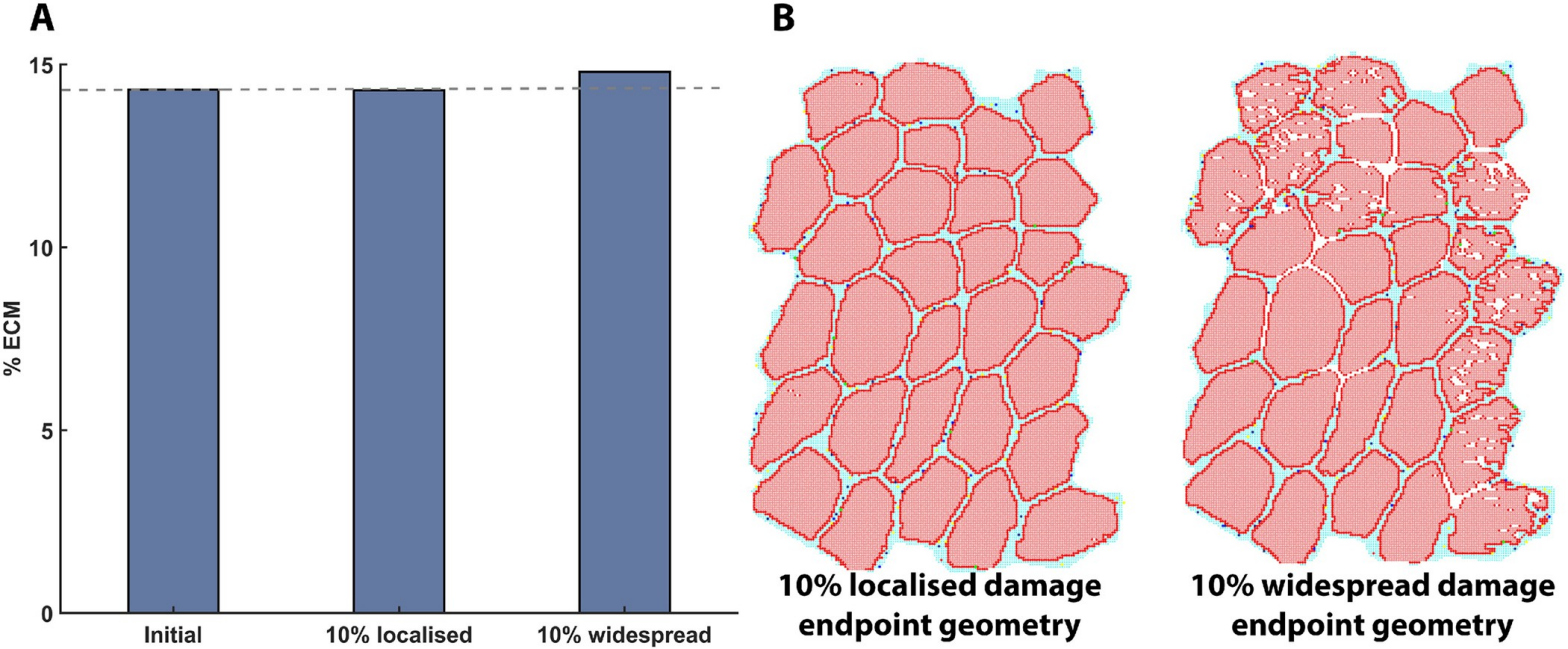
ECM changes during regeneration simulations. (A) Change in percentage ECM at endpoint from initial, for 10% localised and 10% widespread damage, and (B) change in endpoint ABM geometry from starting geometry for 10% localised and 10% widespread damage. Widespread damage leads to gaps in muscle fibres and changes in fibre shape.

The amount of ECM damage was greater in widespread damage due to excess necrosis that occurs in surrounding cells. ECM repair met initial counts throughout all damage levels. ECM was increased from 4318 to 4401 (1.9%) in the 10% widespread damage scenario and was not significantly different between endpoints; however, the change from initial ECM percentage is increased and the endpoint geometry shows marked differences in the repair outcomes (Fig 5).

**Fig 6:**
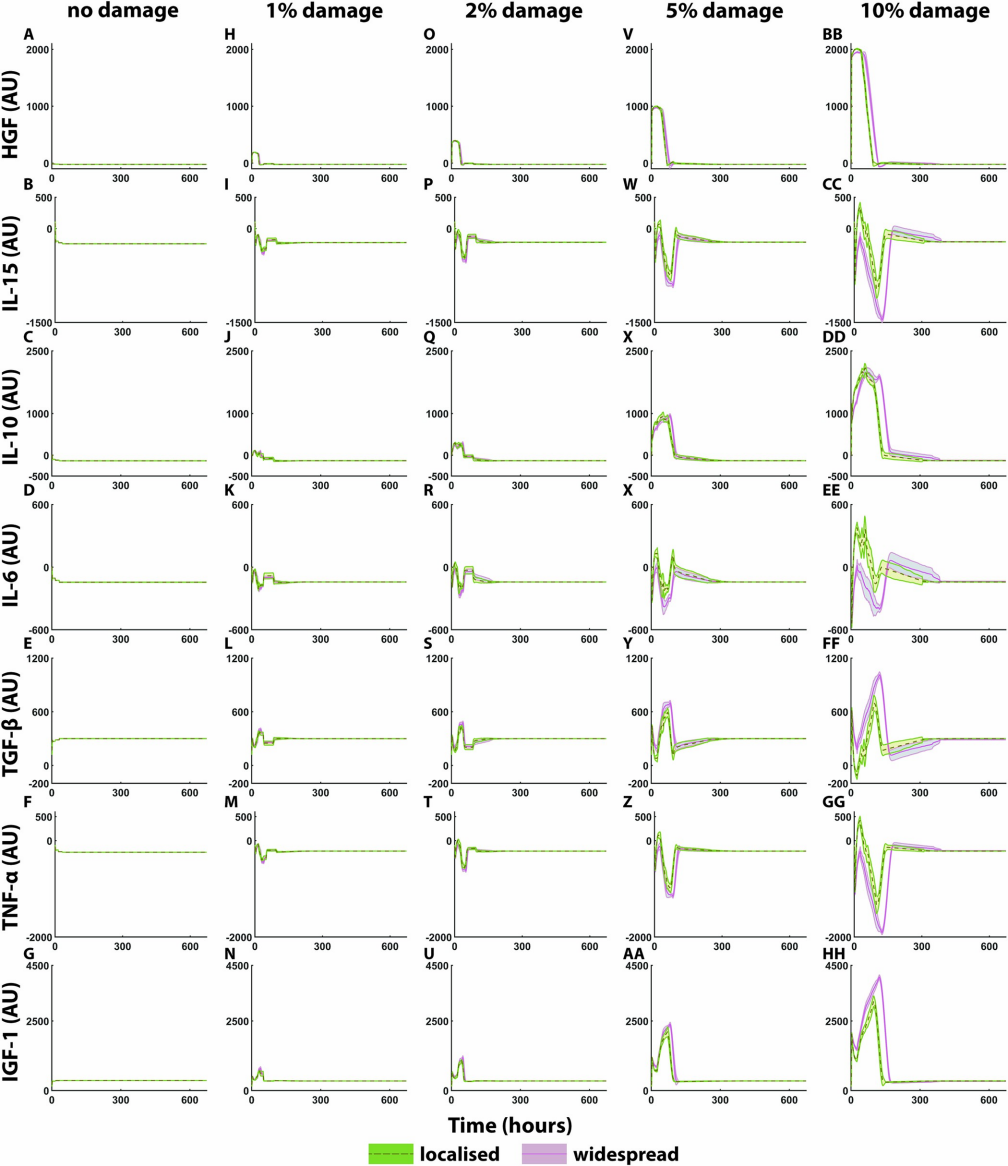
Cytokine and growth factor levels for muscle regeneration over 672 h. (A-G) no damage levels, (H-N) cytokine response following 1% damage, (O-U) 2% damage cytokine response, (V-BB) 5% damage cytokine response, and (CC-II) 10% damage cytokine levels over 672 hours, mean ± SD. In these simulations, 0 represents a starting value or steady state that has been optimised for but is not absolute. The subsequent changes to these values then represent increases or decreases relative to the optimised value.

Cytokine levels were recorded over the simulation time frame in arbitrary units (AU) (Fig 6). In these simulations, 0 represents a starting value or steady state that has been optimised; thus, the graph plots represent the change from baseline rather than absolute concentrations. Initial levels are shown in the 0% damage column (Fig 6A–6G), in response to 1% damage (Fig 6H–6N), 2% damage (Fig 6O–6U), 5% damage (Fig 6V–6BB), and 10% damage (Fig 6CC–6HH). HGF levels increased as damage percentage increased. HGF was initially high after 1% localised damage until 32 h, with a five-hour delay for widespread damage. The duration of elevated HGF increased with damage percentage to 42, 68 and 96 h at 2, 5, and 10% localised damage, respectively. Widespread damage levels had increased duration of elevated HGF (47, 79 and 117 h for 2, 5, and 10% damage, respectively).

IL-15 and IL-6 levels were lower at peak amplitude for 5% and 10% widespread damage compared to localised at 0–166 h, and 0–157 h, respectively. IL-10 levels were similar between widespread and localised damage for each damage percentage; however, at 10% damage IL-10 remained high for 35 hours longer in widespread compared to localised damage. IGF-1 and TGF-β peak levels were higher for widespread damage (2364 at 83 h for 5% damage, 4056 at 125 h for 10% damage for IGF-1, and 700 at 82 h for 5% damage, 1013 at 124 h for 10% for TGF-β) compared to localised (2084 at 69 h for 5% damage, 3208 at 98 h for 10% damage for IGF1, and 637 at 72h for 5% 782 at 101 h for 10% for TGF-β) and this was more prominent at the higher damage percentages of 5 and 10%. In contrast, TNF-α levels were more elevated following localised damage for 5% and 10% damage.

## Discussion

Skeletal muscle regeneration research has previously focused on phenotypes and behaviour of individual cell types, and little prior work has leveraged the power of agent-based modelling to explore an array of cell interactions at play during muscle regeneration. However, it is important to investigate the impact of the multitude of cells in the system and the impact that disease has on the regulation of the regeneration process. In accordance with this, we have presented a mechanobiological model of muscle regeneration following localised and widespread damage that shows similarities to what is observed during chronic myopathies such as DMD and IMs.

Fibril cell counts over time demonstrated that 1, 2, and 5% damage elicited an increase in fibril recovery above the initial levels which is indicative of hypertrophy. Between 5 and 10% damage, there is a threshold damage percentage at which the time for repair is not sufficient to reach the initial counts for widespread damage. 10% widespread damage demonstrated a loss of fibrils and changes in muscle fibre morphology. The slight change in ECM fraction between initial and 10% damage simulations was made more apparent when compared to the decrease in fibril recovery and overall geometry. Fibroblast activity over an extended repair time frame would likely result in a further increase in ECM deposition based on the model output. In DMD histology, fibres lose their regular shape, become split and smaller [71]. Variation in fibre cell diameter is observed in polymyositis as well as increased perimysial and epimysial connective tissue accumulation [72]. These changes are consistent with 10% widespread damage simulation outcomes. Cell behaviour rules were the same under both simulation conditions, and this suggests that emergent behaviour of the simulation arises from geometric differences in damage presentation between the two cases, with the localised repair zones being more concentrated and therefore faster to repair.

Higher peak neutrophil concentration in the localised simulations compared to the widespread simulations is likely due to more ‘efficient’ labelling of damage and subsequent interaction with macrophages. This is seen in other tissues where neutrophils identify damage and promote the phenotypic conversion of macrophages [73]. Macrophage peak numbers in the widespread simulations peak later due to the diffuse nature of the injury, and this effect is compounded by fewer neutrophils which identify repair sites more slowly. Inflammatory cell infiltrate has also been shown to be asynchronous in chronic myopathies compared to a more phasic appearance and removal of inflammatory cells in response to acute, exercise-induced muscle damage [74]. The ABM presented here only differs in the way that damage is seeded, therefore, this approach demonstrates differences in cell numbers over time based solely on the damage localisation mechanism. This suggests that a major factor in impaired or incomplete damage in inflammatory diseases is the diffuse nature of the injury, compared to more localised damage that is observed in muscle following eccentric exercise. Muscle fibre repair cannot simply be described as bigger injury leads to longer repair time. Rather, sufficiently big bouts of damage do not fully recover because of extracellular matrix remodelling by fibroblasts which compete with satellite cells ability to repair contractile fibres [19].

The current model has considered pro-inflammatory M1 macrophages and anti-inflammatory M2 macrophages to be a single agent type with two phenotypes. Macrophages in this model undergo a phenotypic switch after efferocytosis of damaged tissue. The principles that distinguish between the two phenotypes in human muscles are not detailed enough to be able to programme distinct agent classes with distinct rules. Future models should aim to incorporate this level of detail into agent descriptions. Interestingly, the macrophage response was reduced in localised damage simulations and required less time to clear debris. There was greater neutrophil recruitment in localised damage compared to widespread, and this may account for the faster clearance time of damaged tissue, as the neutrophils actively control the acute inflammatory response through removal of damaged tissue and release of cytokines that modulate the subsequent cell behaviour [75]. Sciorati et al. [76] describe the clearance of dying cells in skeletal muscle as critical to muscle homeostasis. The localised damage simulations demonstrated efficient clearance, while the widespread damage simulations had delayed clearance. It is well known that delayed or insufficient inflammatory clearance, for example due to anti-inflammatory medication, has marked effects on the outcome of regeneration as a whole by attenuating recruitment of SCs [77]. In addition, early ablation of macrophages in mice leads to decreases in anti-inflammatory macrophages which are crucial to robust repair [76]. Gaetano et al. [78] have shown that histopathology of polymyositis and inclusion body myositis demonstrate prolonged mononuclear cell infiltrate [72,79], which is also reflected in widespread damage simulations.

Satellite cell numbers and their level of recruitment dictate the speed at which new fibrils are laid down given adequate clearance of damage. Literature values of SC counts differ greatly between 0 and 72 h [9,19,80], with the rate of recruitment not well known in this model. These simulations suggest that SC recruitment scales with damage, but SC time course data for healthy and diseased muscle at various levels of damage has not been recorded. At all localised damage levels, the actions of SCs were sufficient to restore the fibril count, and a fraction of the SCs returned to quiescence. In 10% widespread damage the total depletion of the active SC pool was a surprising outcome. It is important to recognise that in studying the modality of damage alone, other factors, such as satellite cell recruitment in disease, were not tested. SCs are initially upregulated in DMD but then decrease in number of asymmetric divisions, reducing the number of myogenic progenitor cells [81]. Adding in other chronic characteristics of disease is an important consideration given that in DMD, the absence of dystrophin leads to loss of polarity in SCs and therefore a decreased rate of progenitor cell proliferation and overall muscle regeneration [52,82,83].

Hypertrophy is expected in response to mechanically localised damage such as in exercise, with increases of 1% per month when cumulative gains for 12-week resistance training cycles are adjusted [13]. However, it is important to be able to estimate the amount of fibril damage that occurs following a single bout of exercise. While the percentage of fibres with membrane disruption or percentage of sarcomeres with z-disk disruption is often reported with numbers ranging from 22–76% of fibres and 60% of sarcomeres, respectively [84,85], the number of fibrils that are damaged within a muscle bundle or that percentage of a fibre that is damaged is not reported. This study used damage percentages of 1, 2, 5 and 10%, with 10% being a considerable insult to muscle. Our model was not able to discern between 1, 5 and 10% damage as representing an appropriate level of damage, as they all result in roughly 1% increase in fibril count that was expected as a result of muscle repair [13]. However, during simulations with 5% damage applied across all fibril pixels, the model was able to demonstrate that 76% of all fibres (23 out of 30) in the geometry showed some amount of damage, which is comparable to that shown in the literature. The 10% damage simulation represents a more severe scenario in terms of exercise-induced damage, and it is likely that higher percentages would be from injured tissue. Additionally, while the widespread damage simulations recreated outputs that are associated with chronic myopathies, the initial seeding conditions would need to be altered to represent disease conditions. In future disease simulations, the strains imposed by eccentric lengthening would be added to any damage from the progression of chronic myopathies that exist in that tissue. This would mean an increased overall percentage of damage in the widespread model compared to localised, and the differences between the simulation states would likely be obvious at lower levels of user-prescribed damage. The end agent-based model tissue state for chronic simulations would then be used to seed starting conditions of the subsequent repair cycle to illustrate the muscle environment more accurately in diseases with persistent widespread damage.

There is limited information on baseline cytokine and growth factor concentrations that can markedly alter cell response to damage, as well as chemical regulation of the environment. This study was limited in selection of cytokines, and these were based on those most studied in skeletal muscle. Cytokine alterations in IMs include increased IL-10 and TNF-α from mononuclear cells, and decreased TGF-β [79]. The latter two outcomes are not reflected in these simulations, but the cytokine profiles for IMs differ from those commonly studied for typical muscle repair; thus, inclusion of disease specific cytokines is an important consideration. Though empirical studies of cytokine levels over time are available, many of the datapoints in these studies are not suited for comparison and seeding of this model because of the type of injury, the acute timepoints, and measurement differences (S1 Table). For that reason, an optimisation algorithm was employed despite it being an underdetermined system.

The FEM used to seed fibril damage in the ABM demonstrated the contribution of realistic fibre geometry to the generated strain values. Fibres with less rounded (more angular) geometry had higher strain values, consistent previous muscle FEM that have demonstrated high dependence of strain localisation on the underlying geometry [86,87]. In addition to this, fibres with thin ECM between them also had higher strain values as they pulled the surrounding ECM in the direction of the displacement. Interestingly, the strain pattern in the FEM model was similar at 1, 2, 5 and 10%, but with varying magnitude (S1 Fig) so in this case using a single FEM with damage percentages for the ABM simplified the modelling process. Since muscle fibre bundle cross section is relatively consistent between muscles under typical conditions [87], the FEM and ABM represents a generalisable section of tissue. However, further analysis is required for modelling cross-sections from pathological fibre bundles, which should be performed for the widespread damage simulation. Consideration should also be given to the slice thickness used in the ABM. Altering the slice thickness to include increased numbers of SCs and fibroblasts would have faster cell dynamics however, the approximate peak cell counts and temporal dynamics for the increased thickness would have to be tuned to match with empirical data, therefore the simulations are expected to have the same relative differences between widespread and localised damage scenarios. A 3D ABM would offer more realistic cell migration along the length of a cell to regenerating zones for repair [39].

Cytokine and growth factor profiles were presented in this study in levels relative to initial; therefore, it is difficult to compare these to existing data on muscle repair where much of the available data represent the first 24 h time frame. HGF is known to be released from the ECM and activates quiescent SCs [50,88]. It also regulates the transition of M1 macrophage to anti-inflammatory M2 macrophages [28]. This transition seems to be faster in localised injury conditions, and prolonged TGF-β expression in the 10% widespread simulation is also reflective of macrophage secretions. M2a macrophages are increased in ageing muscle that can lead to increased fibrosis [89]. Zhao et al. (2008) have shown that deletion of myostatin, the TGF-β family myokine, in mouse models of DMD led to reductions in fibrosis [90]. Overall, these studies demonstrate the need for tight regulation of cytokine and growth factors in response to muscle injury.

The purpose of this model at the outset was to evaluate whether the mode of injury alone influenced the outcomes of muscle repair. However, the simulation of non-localised widespread damage looked similar to disease presentation. In future, the widespread model may be tuned to look at disease phenotypes with parameters that are tuned to the nature of specific diseases. In chronic myopathies, mechanical damage occurs alongside inflammatory damage, and this should be explored in future iterations of this model at physiologically relevant levels of damage. Empirical measurements from healthy and pathological tissues that are useful to validate the results of the model include gross scale endpoint muscle volume changes and cell scale histological changes in average fibre CSA after exercise. Additionally, concentrations of cells, growth factors and cytokines at both the mid- and end-repair cycle stages offer a way to directly compare model outputs with experimental findings. The emergent outcomes of the model can then be compared to empirical measurements to assess the feasibility of the model. These experimental validations are a way in which empirical research can guide computational systems biology and vice versa, with the former informing useful model creation and the latter leading to more specific hypothesis testing to uncover mechanisms that a crucial to skeletal muscle repair and remodelling in different damage conditions.

## Conclusions

Appropriate mechanotransduction of external forces is important for cellular behaviour in skeletal muscle. Bouts of widespread inflammatory damage, such as that in disease, lead to longer periods of inflammatory cell invasion and attenuated repair, which is more apparent at high percentages of damage. Overall changes to ECM and fibril counts following widespread damage suggest that the decrease in contractile tissue is a negative outcome of muscle function and repair. This study used *in silico* mechanobiological modelling to investigate the differences in skeletal muscle regeneration between mechanically mediated and widespread inflammatory damage. There were stark differences in outcomes between mechanical injury and inflammatory damage following high levels of damage, indicating fundamental differences between the way these injury mechanisms influence regeneration. The outcomes observed here recapitulated features of muscle disease such as altered muscle fibre morphology and increased collagen deposition. This study explores the mediators of muscle regeneration during exercise-induced and inflammatory muscle damage, and may be built upon in research exploring therapeutic and pharmaceutical targets to mitigate the muscle degeneration in chronic muscle diseases such as inflammatory myopathies and DMD.

## Supporting information

S1 Fig Muscle fibre FEM results at 1, 2 and 5% displacement, obtained from the 10% displacement FE simulation used to seed the ABM. The areas of high strain (red) across the muscle fibre bundle are consistent at different displacement percentages.
(TIF)

S1 Table Brief description of studies investigating cytokine concentrations over time in human tissues. These studies vary in timepoints, mode of injury, and analysis, and are therefore not directly suitable for seeding agent-based models.
(DOCX)
