# Supplementary material for: Delayed skeletal muscle repair following inflammatory damage in simulated agent-based models of muscle regeneration
Source: PLoS Comput Biol. 2023 Apr 6;19(4):e1011042. doi: 10.1371/journal.pcbi.1011042 (PMC10128985; doi:10.1371/journal.pcbi.1011042)
Supplement: S1 Table — These studies vary in timepoints, mode of injury, and analysis, and are therefore not directly suitable for seeding agent-based models. (DOCX) [file pcbi.1011042.s002.docx]

Supplementary Table 1 Brief description of studies investigating cytokine concentrations over time in human tissues. These studies vary in timepoints, mode of injury, and analysis, and are therefore not directly suitable for seeding agent-based models.

| **Reference** | **type of injury** | **analysis** | **time points (hours post injury)** | **units** | **species** | **cytokine(s)** |
| --- | --- | --- | --- | --- | --- | --- |
| Kang et al, 2018 | cardiotoxin | tissue mRNA | 0, 168 | fold change | human, mouse | IL-15 |
| Ostrowski et al 1998 | Running training | tissue mRNA and plasma concentrations | pre, 0, 2 | fold change, pg/mL | human | IL-6, TNF-a |
| Steensberg et al 2002 | knee extensor exercise | arterial plasma, tissue mRNA | pre, during, 180 | fold change, pg/mL | human | TNF-a IL-6 |
| O'Reilly et al 2008 | eccentric contractions | western blot, serum HGF | pre, 4, 24, 72, 120 | pg/mL, Averaged HGF normalised to Actin | human | HGF |
| Neilsen et al 2007 | strength training | western blot, tissue mRNA | pre, 6, 24, 48 | mRNA fold change, relative protein levels | human | IL-15 |
| Kazemi et al 2015 | resistance exercise | plasma | pre, 24 | pg/mL | human | IL-10, IL-6 |
